# Diabetes Mellitus and Albuminuria

**Published:** 1882-03

**Authors:** 


### Diabetes Mellitus and Albuminuria.

According to Frerich, in true diabetes albuminuria is not frequent, nephritis is rare, and occurs only in certain conditions. Out of 316 cases of diabetes mellitus which Frerich partly observed for ten, twelve, and even fifteen or sixteen years, there were only sixteen cases of nephritis. Cases in which small quantities of albumen were occasionally found in the urine were never pure and simple cases, since it could be proved in most of them that other processes were involved which of themselves could produce nephritis. Of sixteen cases there were only three in which no co-operating cause could be suggested. The author concludes that the general belief that the increased action of the kidneys in diabetes mellitus leads to albuminuria and nephritis is erroneous. According to the author's postmortem examinations, which comprise more than fifty cases, true nephritis is hardly ever met with in diabetes.—*Boston Med. and Surgical Journal.*

